# Magnetic Guinier law

**DOI:** 10.1107/S2052252519016439

**Published:** 2020-01-01

**Authors:** Andreas Michels, Artem Malyeyev, Ivan Titov, Dirk Honecker, Robert Cubitt, Elizabeth Blackburn, Kiyonori Suzuki

**Affiliations:** a Department of Physics and Materials Science, University of Luxembourg, 162A Avenue de la Faïencerie, L-1511 Luxembourg, Grand Duchy of Luxembourg; b Institut Laue-Langevin, 71 avenue des Martyrs, F-38042 Grenoble, France; c Division of Synchrotron Radiation Research, Department of Physics, Lund University, SE-22100 Lund, Sweden; d Department of Materials Science and Engineering, Monash University, Clayton, Victoria 3800, Australia

**Keywords:** small-angle neutron scattering, Guinier law, magnetic materials, micromagnetics, nanoscience, magnetic scattering, ferromagnets, anisotropy

## Abstract

Small-angle scattering of X-rays and neutrons is a routine method for the determination of nanoparticle sizes. The so-called Guinier law represents the low-*q* approximation for the small-angle scattering curve from an assembly of particles. The Guinier law has originally been derived for nonmagnetic particle-matrix-type systems and it is successfully employed for the estimation of particle sizes in various scientific domains (*e.g.* soft-matter physics, biology, colloidal chemistry, materials science). An important prerequisite for it to apply is the presence of a discontinuous interface separating particles and matrix. Here, the Guinier law is introduced for the case of magnetic small-angle neutron scattering and its applicability is experimentally demonstrated for the example of nanocrystalline cobalt. It is well known that the magnetic microstructure of nanocrystalline ferromagnets is highly nonuniform on the nanometre length scale and characterized by a spectrum of continuously varying long-wavelength magnetization fluctuations, *i.e.* these systems do not manifest sharp interfaces in their magnetization profile. The magnetic Guinier radius depends on the applied magnetic field, on the magnetic interactions (exchange, magnetostatics) and on the magnetic anisotropy-field radius, which characterizes the size over which the magnetic anisotropy field is coherently aligned into the same direction. In contrast to the nonmagnetic conventional Guinier law, the magnetic version can be applied to fully dense random-anisotropy-type ferromagnets.

## Abstract 2

The Guinier law for magnetic SANS on bulk ferromagnets is introduced and applied to the analysis of nanocrystalline cobalt. The magnetic-field-dependent Guinier radius reflects the characteristic microstructural size and depends on the magnetic interactions.

## 1. Introduction   

The determination of particle sizes is one of the most important daily tasks in many branches of the natural sciences. While particle sizes in the micrometre regime and above can be conveniently determined using *e.g.* optical microscopy, the size of nanoparticles (with *D* ≃ 1 − 100 nm) requires scanning and/or transmission electron microscopy, or other scattering methods such as X-ray or neutron scattering. While the former techniques inherently suffer from low statistics, the latter techniques have the advantage of providing statistically averaged information over a large number of particles. Small-angle scattering, using either X-rays or neutrons, is one of the most popular methods for analyzing structures on the mesoscopic length scale, embracing a broad range of research topics from condensed-matter and soft-matter physics, to physical chemistry, biology, and materials science (Svergun *et al.*, 2013 ▸).

The well known Guinier law describes the elastic small-angle scattering of X-rays and neutrons near the origin of reciprocal space (Guinier & Fournet, 1955 ▸). When the scattering is from a dilute and monodisperse set of objects (particles) with sharp interfaces, then the macroscopic differential scattering cross-section dΣ/dΩ in the limit of low-momentum transfers *q* < 1.3/*R*_*G*_ can be expressed as (Porod, 1982 ▸; Feigin & Svergun, 1987 ▸) 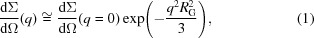
where the forward scattering cross-section (dΣ/dΩ)(0) is proportional to the squared total excess scattering length of the particle and *R*_G_ denotes the particle’s radius of gyration. Equation (1) is valid for arbitrary particle shapes. From a Guinier plot, ln(dΣ/dΩ) versus *q* ^2^, one can determine *R*_G_, which is related to the particle size, *e.g.* *R*_G_^2^ = (3/5)*R* ^2^ for a sphere of radius *R*. The Guinier law is of outstanding importance for the analysis of small-angle scattering data, particularly at the first stage of the data analysis.

From the foregoing discussion it is clear that the Guinier law has been derived for nonmagnetic particle-matrix-type assemblies in the context of the early theoretical developments of the technique of small-angle X-ray scattering (SAXS) (Guinier & Fournet, 1955 ▸). Therefore, its application to magnetic materials, which is the subject of the present article, should be considered with special care; for instance, the Guinier law is certainly applicable to systems consisting of saturated and homogeneous magnetic particles in a nonmagnetic and homogeneous matrix or, likewise, to pores in a saturated matrix. In this context, we refer to the article by Burke (1981 ▸) who investigated the influence of magnetic shape anisotropy on the Guinier law of fine ferromagnetic single-domain particles. By contrast, when the sample is inhomogeneously magnetized on the nanometre length scale, *i.e.* when the magnitude and orientation of the magnetization vector field **M** varies continuously with the position **r** inside the material, then a central assumption underlying the Guinier law – namely that of domains (particles) separated by discontinuous interfaces from the matrix – is violated. Equation (1), with a constant and field-independent *R*_G_, does not then describe the low-*q* region of the magnetic small-angle neutron scattering (SANS) cross-section. Intuitively, it may be clear from the previous considerations that an effective magnetic Guinier radius is expected to depend on the applied magnetic field as well as on the magnetic interactions (*e.g.* exchange, anisotropy, magnetostatics). In the following we derive the magnetic Guinier law and provide an analysis of experimental SANS data of nanocrystalline Co.

This article is organized as follows: Section 2 furnishes the details of the SANS experiment; Sections 3 and 4 introduce the unpolarized SANS cross-section, the theoretical background in terms of micromagnetic theory, and the magnetic Guinier law; Section 5 presents and discusses the experimental results of the magnetic Guinier analysis on nanocrystalline Co; and Section 6 summarizes the main results of this study. In the Supporting information for this article, the two- and one-dimensional total SANS cross-sections and a graphical representation of the relative error of the magnetic Guinier approximation are featured.

## 2. Experimental   

**Figure 1:**
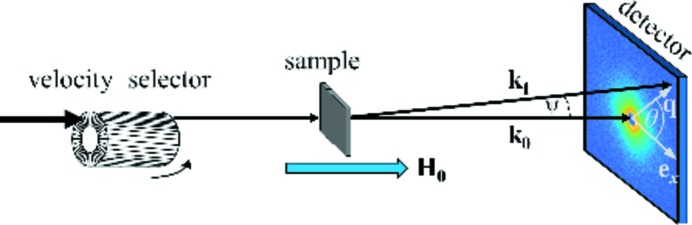
Sketch of the neutron setup. The external magnetic field **H**_0_ ∥ **e**_*z*_ is applied parallel to the wavevector **k**_0_ of the incident neutrons. In the small-angle approximation, the momentum-transfer or scattering vector, **q** = **k**_1_ − **k**_0_, varies in the plane perpendicular to **k**_0_, *i.e.* **q** ≅ {*q*_*x*_, *q*_*y*_, 0} = *q* {cos θ, sin θ, 0}. The magnitude of **q** for elastic scattering is given by *q* = (4π/λ) sin (ψ/2), where λ denotes the mean neutron wavelength (selected by the velocity selector) and ψ is the scattering angle. The angle θ specifies the orientation of **q** on the two-dimensional detector.

The SANS experiment was conducted at 300 K using the instrument D11 at the Institut Laue-Langevin, Grenoble. We used unpolarized incident neutrons with a mean wavelength of λ = 6 Å and a bandwidth of Δλ/λ = 10% (FWHM). The instrument offers access to a low *q* range of 0.016 nm^−1^ ≲ *q* ≲ 0.2 nm^−1^ with the two-dimensional position-sensitive detector placed at a distance of 38.5 m from the sample position. The external magnetic field **H**_0_ (with μ_0_ *H*_0_^max^ = 16.5 T where μ_0_ is the permeability of free space and *H*_0_^max^ is the maximum external magnetic field component) was applied parallel to the wavevector **k**_0_ of the incoming neutron beam (see Fig. 1 ▸ for a sketch of the neutron setup).

The nanocrystalline Co sample under study was synthesized by means of pulsed electrodeposition. We emphasize that this particular sample has been extensively studied in the past using magnetometry, wide-angle X-ray diffraction, and unpolarized and spin-polarized SANS (*e.g.* Michels *et al.*, 2000 ▸, 2003 ▸, 2014 ▸; Weissmüller *et al.*, 2001 ▸; Honecker *et al.*, 2011 ▸; Mettus & Michels, 2015 ▸). It is also important to note that it is a fully dense polycrystalline bulk metal with a nanometre grain size [average crystallite size: *D* = 9.5 ± 3.0 nm (Weissmüller *et al.*, 2001 ▸)], not nanoparticles in a matrix. The SANS sample consisted of a single circular disk. Based on the thickness (80 µm) and the diameter (2 cm) of the disk, we computed a demagnetizing factor of *N* ≅ 0.994 for the case that **H**_0_ is parallel to the surface normal of the sample (Osborn, 1945 ▸), in agreement with the **k**_0_ ∥ **H**_0_ scattering geometry of the SANS experiment. Using the value of the saturation magnetization of Co, μ_0_ *M*_s_ = 1.80 T (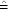
1434 kA m^−1^), this results in a demagnetizing field of μ_0_ *N* *M*_s_ ≅ 1.789 T for a fully saturated sample. In the following all the reported field values are corrected for demagnetizing effects. To reduce the influence of inhomogeneous demagnetizing fields at the outer perimeter of the circular sample, the neutron beam was collimated to a diameter of 0.8 cm. The neutron transmission was larger than 90% in all measurements, indicating a negligible influence of multiple scattering.

## 3. SANS cross-section and micromagnetic theory   

When the external magnetic field **H**_0_ ∥ **e**_*z*_ (applied field direction **H**_0_ defines the **e**_*z*_ direction of a cartesian coordinate system) is applied parallel to the wavevector **k**_0_ of the incoming neutron beam (Fig. 1 ▸), the unpolarized elastic differential SANS cross-section dΣ/dΩ at momentum-transfer vector **q** equals that given in Mühlbauer *et al.* (2019 ▸). 
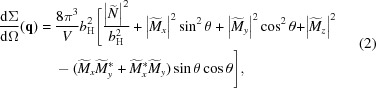
where *V* is the scattering volume and the constant *b*_H_ = 2.91 × 10^8^ A^−1^m^−1^ relates the atomic magnetic moment μ_a_ to the atomic magnetic scattering length *b*_m_ ≅ *b*_H_μ_a_ (in small-angle approximation). 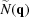 and 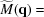 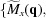 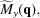 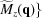 represent, respectively, the Fourier transforms of the nuclear scattering-length density *N*(**r**) and the magnetization **M**(**r**) = {*M*_*x*_(**r**), *M*_*y*_(**r**), *M*_*z*_(**r**)}, the superscript ‘*’ refers to the complex-conjugated quantity, and θ denotes the angle between **q** and **e**_*x*_. Note that in the small-angle approximation the component of **q** along the incident beam (**k**_0_ ∥ **e**_*z*_) is negligible compared with the other two components, such that **q** ≅ {*q*_*x*_, *q*_*y*_, 0}. This emphasizes the fact that SANS predominantly probes correlations in the plane perpendicular to **k**_0_.

Further analysis of the magnetic SANS cross-section [equation (2)] requires expressions for the magnetization Fourier amplitudes 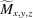. In the works by Honecker & Michels (2013 ▸) and Michels *et al.* (2016 ▸) a quite general theory of magnetic SANS based on the continuum theory of micromagnetics has been developed. In the following we sketch the main ideas of the micromagnetic SANS theory in order to achieve a self-contained presentation. The approach considers two origins of spin misalignment. (i) Spatial nanometre-scale variations in the orientation and/or magnitude of the magnetic anisotropy field **H**_p_(**r**) (*e.g.* at a grain boundary in a single-phase nanocrystalline ferromagnet). Such anisotropy-field fluctuations give rise to torques on the magnetization **M** and result in a concomitant deviation of **M** from the mean magnetization direction (given by a large applied field). (ii) Spatial variations of the saturation magnetization *M*_s_(**r**) give rise to local magnetostatic stray fields (*e.g.* at a particle-matrix interphase in a nanocomposite), which also result in a magnetic SANS contrast. This scenario is adapted to the inhomogeneous magnetic microstructure which is found in many polycrystalline magnets.

The micromagnetic theory takes into account the isotropic and symmetric exchange interaction, magnetic anisotropy, as well as the Zeeman and magnetodipolar interaction energies. As detailed in the pertinent textbooks  (Brown, 1963 ▸; Aharoni, 1996 ▸; Kronmüller & Fähnle, 2003 ▸; Kronmüller & Parkin, 2007 ▸), variational calculus leads to a set of nonlinear partial differential equations for the equilibrium magnetization configuration **M**(**r**). For the static case, the equations of micromagnetics (so-called Brown’s equations) can be conveniently expressed as a balance-of-torques equation,
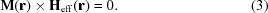
Equation (3) expresses the fact that at static equilibrium the torque on the magnetization **M**(**r**) caused by an effective magnetic field **H**_eff_(**r**) vanishes at each point **r** inside the material. The effective field is obtained as 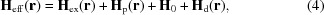
where **H**_ex_(**r**) = *l*_M_^2^ Δ**M**(**r**) represents the exchange field (with Δ the Laplace operator), **H**_p_(**r**) is the magnetic anisotropy field, **H**_0_ is a uniform applied magnetic field, and **H**_d_(**r**) denotes the magnetostatic or magnetodipolar interaction field. The magnetostatic exchange length *l*_M_ = [2*A*/(μ_0_ *M*_s_^2^)]^1/2^ is of the order of a few nanometres for many magnetic materials [*l*_M_ ≃ 3–10 nm (Kronmüller & Fähnle, 2003 ▸)], *A* is the exchange-stiffness constant and *M*_s_ is the saturation magnetization. Then, in the approach-to-saturation regime, the micromagnetic equations can be linearized, and closed-form expressions for the magnetization Fourier components 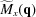 and 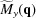 can be obtained [see Honecker & Michels (2013 ▸) and Michels *et al.* (2016 ▸) for details].

Using the results for 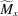 and 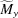, the unpolarized elastic SANS cross-section dΣ/dΩ in the parallel scattering geometry [equation (2)] can be expressed in compact form as 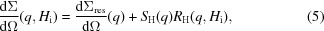
where the (nuclear and magnetic) so-called residual SANS cross-section, 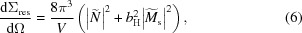
is measured at complete magnetic saturation (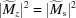), and the remaining spin-misalignment SANS cross-section 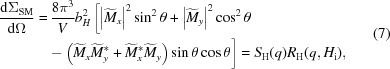
describes the purely magnetic small-angle scattering caused by the misaligned spins with related Fourier amplitudes 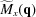 and 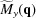 [compare with equation (2)]. Since the magnetic Guinier law is related to the spin-misalignment scattering, it is necessary to separate the total dΣ/dΩ into dΣ_res_/dΩ and dΣ_SM_/dΩ [equation (5)]. In the analysis of experimental data, dΣ_res_/dΩ can be measured at a saturating applied field and subtracted from the dΣ/dΩ at lower fields to obtain the field-dependent dΣ_SM_/dΩ.

The quantity *S*_H_ denotes the anisotropy-field scattering function, which is proportional to the magnitude square of the Fourier transform 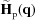 of the magnetic anisotropy field **H**_p_(**r**), *i.e.* 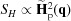. This function contains information on the strength and spatial structure of the magnetic anisotropy field. In the approach-to-saturation regime, which is the validity range of the micromagnetic SANS theory, *S*_H_ is independent of the applied magnetic field. We further note that for a statistically isotropic material *S*_H_ depends only on the magnitude *q* of the scattering vector **q**, not on its orientation (see below). The dimensionless micromagnetic response function *R*_H_ depends on *q* as well as on the internal magnetic field *H*_i_ = *H*_0_ − *N* *M*_s_, where *N* denotes the demagnetizing factor. More specifically (**k**_0_ ∥ **H**_0_), 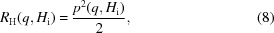
where the dimensionless function, 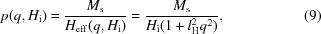
depends on the effective magnetic field *H*_eff_(*q*, *H*_i_) [not to be confused with **H**_eff_(**r**) in equation (3)] and on the micromagnetic exchange length, 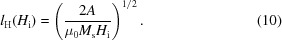
The quantity *l*_H_ characterizes the field-dependent size of perturbed regions around microstructural defects, and, as we will see below, it is this quantity which renders the magnetic Guinier radius field dependent. By inserting typical values for the material parameters of Co [*A* = 2.8 × 10^−11^ J m^−1^ and μ_0_ *M*_s_ = 1.80 T (Michels & Weissmüller, 2008 ▸)], it is seen that the exchange length *l*_H_ varies between about 200–2 nm when the internal field is changed between 0.001–10 T. This length scale falls well into the resolution regime of the SANS technique.

## 4. Magnetic Guinier law   

In order to derive a Guinier expression for magnetic SANS, analogous to equation (1), we look in the following for the low-*q* behavior of the spin-misalignment SANS cross-section dΣ_SM_/dΩ = *S*_H_(*q*)*R*_H_(*q*, *H*_i_) [equation (7)]. The sample volume which is probed by the neutrons typically contains many defects (*e.g.* crystallites separated by grain boundaries), each one having a different orientation and/or magnitude of the magnetic anisotropy field. To obtain a low-*q* approximation for 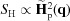, we make the assumption that the total magnetic anisotropy field of the sample, **H**_p_(**r**), is the sum of the anisotropy fields of the individual defects *i* (Weissmüller *et al.*, 2001 ▸, 1999 ▸) *i.e.*

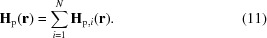
This decomposition also applies to the Fourier transform 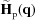 of **H**_p_(**r**), *i.e.*

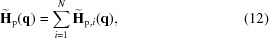
so that 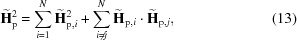
where we have assumed that the 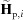 are real-valued quantities. If the 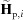 of the individual defects are statistically uncorrelated (random anisotropy), then terms 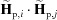 with *i* ≠ *j* take on both signs with equal probability. Consequently, the sum over these terms vanishes and 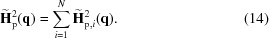
Equation (14) suggests that 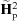, and hence 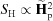, can be computed for an arbitrary arrangement of defects once the solution for the single-defect case 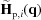 is known. This can, for example, be accomplished for an idealized nanocrystalline ferromagnet, where the crystallites (acting as magnetic defects) have random crystallographic orientation and where the anisotropy field arises exclusively from the magnetocrystalline anisotropy. Because each grain is a single crystal, the anisotropy field in the grain is a constant vector, *i.e.* **H**_p, *i*_ ≠ **H**_p, *i*_(**r**), and the anisotropy field Fourier amplitude is obtained by the following form-factor integral (Weissmüller *et al.*, 2001 ▸): 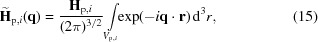
where the integral extends over the volume of grain *i*. For the example of a spherical grain shape [*V*_p, *i*_ = (4π/3)*R*_*i*_^3^], we obtain the well known result that 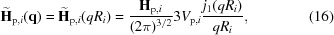
where *j*_1_(*z*) denotes the spherical Bessel function of the first order.

The square of equation (16) is identical, except for the prefactor, to the nuclear SANS cross-section of an array of noninterfering spherical particles, and general asymptotic results at small and large *q* are therefore immediately transferable; in particular, the Guinier approximation relates 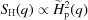 at small-scattering vectors to the radius of gyration *R*_GH_ of the magnetic anisotropy field, according to Weissmüller *et al.* (2001 ▸), 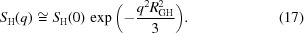
Similar to nuclear SANS and SAXS, where *R*_G_ is a measure for the particle size, *R*_GH_ deduced from *S*_H_ may be seen as a measure for the size of regions over which the magnetic anisotropy field **H**_p_(**r**) is homogeneous. For the special case of an idealized nanocrystalline ferromagnet (random anisotropy and magnetocrystalline anisotropy only), *R*_GH_ is closely related to the crystallite size.

Equation (17) can be combined with the corresponding small-*q* result for the response function [equation (18)]. Taylor expansion of *R*_H_ around *q* = 0 yields: 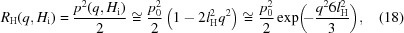
where *p*_0_ = *p*(*q* = 0) = *M*_s_/*H*_i_ [compare with equation (9)]. Inserting equations (17) and (18) into dΣ_SM_/dΩ = *S*_H_ *R*_H_, we have 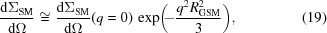
where 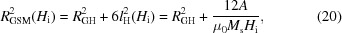
represents the magnetic field-dependent Guinier radius. This relation provides a means to determine the exchange constant *A* from field-dependent SANS measurements. Note that 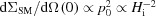 [compare with equation (9)]. The observation that *R*_GSM_ depends on *R*_GH_ and on the micromagnetic exchange length *l*_H_ is a manifestation of the fact that the magnetic microstructure in real space (for which *R*_GSM_ is representative) corresponds to the convolution of the nuclear grain microstructure (*R*_GH_) with field-dependent micromagnetic response functions (*l*_H_).

Up to now we have only discussed the magnetic Guinier approximation for the parallel scattering geometry (**k**_0_ ∥ **H**_0_), where 2π-averaged magnetic SANS data can be used for the analysis in terms of equation (19). In the perpendicular geometry (**k**_0_ ⊥ **H**_0_) an additional scattering term *S*_M_ *R*_M_, related to magnetostatic fluctuations, appears in dΣ_SM_/dΩ, which complicates the discussion. Two comments are then in place. (i) Since 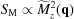, the *S*_M_ *R*_M_ contribution to dΣ_SM_/dΩ can be neglected for single-phase ferromagnets, where fluctuations in the saturation magnetization, *M*_s_, are weak. (ii) Inspection of the expression for the magnetostatic response function *R*_M_ in the perpendicular geometry [equation (29) in Honecker & Michels (2013 ▸)] shows that this function vanishes by taking an average of the two-dimensional dΣ_SM_/dΩ along θ = 0° (or θ = 180°), while the corresponding *R*_H_(θ = 0°) = *p* ^2^ [equation (28) in Honecker & Michels (2013 ▸)] is almost equal (besides a factor of 1/2) to *R*_H_(θ = 0°) = *p* ^2^/2 in the parallel geometry. In other words, these considerations imply that the magnetic Guinier law equation [equation (19)] can also be employed to analyze (θ = 0°) sector-averaged data in the **k**_0_ ⊥ **H**_0_ geometry.

## 5. Experimental results and discussion   

**Figure 2:**
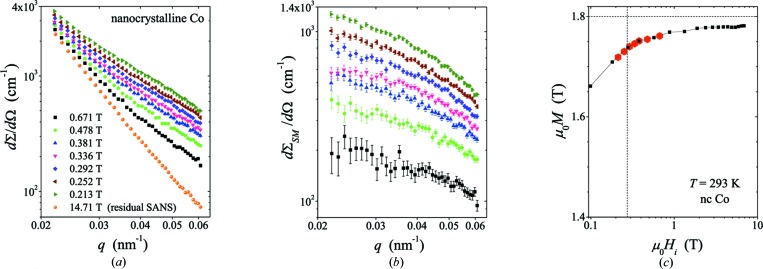
(*a*) 2π-azimuthally-averaged total nuclear and magnetic SANS cross-section dΣ/dΩ of nanocrystalline Co versus momentum transfer *q* at a series of internal magnetic fields (see inset) (log–log scale) (**k**_0_ ∥ **H**_0_). (*b*) Corresponding spin-misalignment SANS cross-section dΣ_SM_/dΩ obtained by subtracting the dΣ/dΩ data at 14.71 T [orange data points in (*a*)] from the dΣ/dΩ at lower fields. (*c*) Magnetization curve of nanocrystalline Co (only the upper-right quadrant is shown). The large red data points indicate the internal-field values where the SANS data were taken. The horizontal dashed line indicates the saturation-magnetization value of μ_0_ *M*_s_ = 1.80 T. The vertical dashed line indicates the approach-to-saturation regime (*M*/*M*_s_ ≳ 96%).

As expected, the two-dimensional SANS intensity distributions of the nanocrystalline Co sample are isotropic (θ independent) at all fields investigated. Although the individual scattering contributions to equation (2) are highly anisotropic, which is owing to the trigonometric functions and the magnetization Fourier components 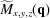, which themselves may depend on the angle θ via the magnetodipolar interaction [compare with Fig. 5 in Michels (2014 ▸)], their sum results in an isotropic (θ independent) dΣ/dΩ for a statistically isotropic grain microstructure (see the Supporting information). This supports the assumption made in the micromagnetic theory of a statistically isotropic grain micro­structure. By contrast, for the perpendicular scattering geometry (**k**_0_ ⊥ **H**_0_), the magnetic SANS cross-section of untextured samples exhibits a variety of angular anisotropies (*e.g.* Löffler *et al.*, 2005 ▸; Bischof *et al.*, 2007 ▸; Michels *et al.*, 2003 ▸). The two-dimensional nuclear and magnetic SANS data were azimuthally averaged over an angle of 2π. To apply equations (19) and (20) to experimental dΣ_SM_/dΩ data [compare with equation (7)], the scattering close to saturation (here at 14.71 T internal field), corresponding to the residual SANS cross-section dΣ_res_/dΩ [equation (6)], needs to be subtracted from the total dΣ/dΩ at lower fields [equation (5)]. The subtraction procedure along with the room-temperature magnetization curve are depicted in Fig. 2 ▸. Besides eliminating the nuclear and the longitudinal magnetic scattering, the subtraction also removes any background scattering contribution.

**Figure 3:**
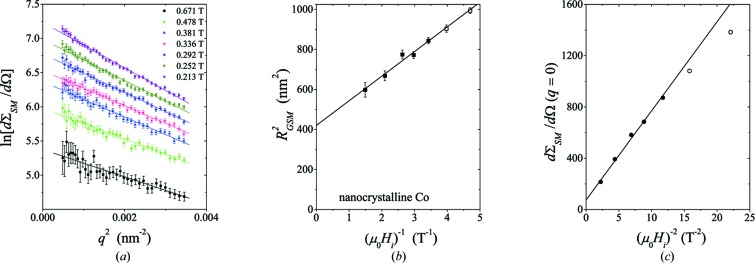
Magnetic Guinier analysis on nanocrystalline Co. (*a*) Guinier plot ln(dΣ_SM_/dΩ) versus *q* ^2^ and fits (solid lines) to equation (19) at selected values of the internal magnetic field (see inset). (*b*) Plot of R_GSM_^2^ versus *H*_i_^−1^ and fit (solid line) to equation (20). In the fitting routine, *R*_GH_ and *A* were treated as adjustable parameters. (*c*) Field dependence of (dΣ_SM_/dΩ) (*q* = 0). The solid line represents  dΣ_SM_/dΩ (*q* = 0) ∝ *H*_i_^−2^. In (*b*) and (*c*), the last two data points (open symbols), corresponding to internal fields of 0.213 and 0.252 T, have been excluded from the fit analysis.

By inspection of Fig. 2 ▸(*c*), we see that the magnetization state of the specimen used in the SANS experiment (indicated by the large red data points) falls well into the approach-to-saturation regime, which is reached for μ_0_ *H*_i_ ≳ 0.27 T(*M*/*M*_*s*_ ≳ 96%, see the discussion below). The shape of dΣ_SM_/dΩ is substantially different to that of dΣ/dΩ, which is owing to the subtraction of the nuclear and saturation scattering [see also the discussion in the work by Bick *et al.* (2013 ▸)]. When the internal field is decreased from 0.671 to 0.213 T, dΣ_SM_/dΩ increases strongly by a factor of ∼6–7 at the smallest momentum transfers (*q*). The strong field dependence of dΣ_SM_/dΩ supports the notion that scattering caused by transversal spin misalignment represents by far the dominant contribution to dΣ/dΩ (see also Fig. 3 in the work by Michels, 2014 ▸). The experimental neutron data in Fig. 2 ▸(*b*) cannot be reproduced by decomposing the cross-section into a set of noninterfering single-domain particles. Careful scrutiny of Fig. 2 ▸(*b*) reveals that the point with the largest curvature in dΣ_SM_/dΩ evolves to larger *q* values with increasing *H*_i_, in agreement with the concomitant decrease of the exchange length *l*_H_ in equation (20).

Fig. 3 ▸ features the magnetic Guinier analysis on nanocrystalline Co. Fig. 3 ▸(*a*) shows the Guinier plots, *i.e.* ln(dΣ_SM_/dΩ) versus *q* ^2^, along with the weighted linear least-squares fits to equation (19), whereas Fig. 3 ▸(*b*) displays the obtained *R* ^2^_GSM_ as a function of *H*_i_^−1^ together with a weighted linear least-squares fit to equation (20). In Fig. 3 ▸(*c*), the field dependence of dΣ_SM_/dΩ (*q* = 0) is displayed. The Guinier plots in Fig. 3 ▸(*a*) reveal that straight-line fits may not be appropriate for the data at the two smallest internal fields of 0.213 and 0.252 T, where an upward curvature becomes visible at the smallest *q*, in contrast to the data at higher field. In line with this observation we see that the dataset in Fig. 3 ▸(*c*) starts to deviate from the expected linear behavior for these two smallest internal fields (open symbols). This discrepancy can be explained with the growing deviations from the small-misalignment approximation for decreasing fields and can be taken as a criterion for the validity range of the approach. Therefore, the two data points at 0.213 and 0.252 T were not taken into account in the Guinier analysis, which yields *R*_GH_ = 20.5 ± 1.2 nm and *A* = (1.5 ± 0.2) × 10^−11^ J m^−1^ [Fig. 3 ▸(*b*)]. The *A* value perfectly fits within the range of values reported in the literature  (Kronmüller & Fähnle, 2003 ▸; Skomski, 2008 ▸), while the *R*_GH_ value corresponds to a spherical particle radius of *R* ≅ 26.5 nm, assuming the relation *R* ^2^_GH_ = (3/5) *R* ^2^, which is valid for monodisperse particles. This value is larger than the average crystallite size of 10 nm (determined by X-ray diffraction), an observation, which can be naturally explained by the presence of a particle-size distribution in our Co sample. It is well known from nuclear SANS theory that a size distribution strongly weighs the *R*_G_ value towards the largest features in the distribution; for instance, for spherical particles and point collimation, *R*_G_^2^ is then related to the ratio of the eighth over the sixth moment of the size distribution (Feigin & Svergun, 1987 ▸; Kostorz, 1982 ▸). Therefore, for the determination of the scaling relation between *R*_GH_ and the average crystallite size, knowledge on the particle-size distribution is required. Lastly, as can be seen in Fig. 3 ▸(*c*), the extrapolated forward-scattering cross-section dΣ_SM_/dΩ (*q* = 0) also obeys the theory prediction and follows the dΣ_SM_/dΩ (*q* = 0) ∝ *H*_i_^−2^ scaling [compare with equation (18)].

The present theory describes the magnetic SANS cross-section of polycrystalline bulk ferromagnets near magnetic saturation, and their low-*q* behavior (magnetic Guinier law). It assumes that the perturbing magnetic anisotropy fields of the individual microstructural defects, which cause a perpendicular magnetization component and hence a contrast for magnetic SANS, vary randomly from defect site to defect site. For the particular case of a nanocrystalline bulk ferromagnet composed of single-crystal grains and atomically-sharp grain boundaries (magnetocrystalline anisotropy only), the characteristic correlation length of the anisotropy-field variation is related to the average crystallite size. Other potential sources of spin inhomogeneity, such as surface (grain-boundary) anisotropy or magnetoelastic anisotropy caused by long-ranged stress fields, are not explicitly included in our theory. Likewise, this approach is not expected to describe the magnetic SANS of inhomogeneously magnetized nanoparticles, which are embedded in a nonmagnetic matrix. For such a microstructure, boundary conditions for the magnetization at the particle–matrix interface must be included in the micromagnetic description of SANS, for which there is currently no analytical solution. This poses a challenge for future studies.

## 6. Conclusions   

Based on the continuum theory of micromagnetics, we have introduced the magnetic Guinier law for random-anisotropy-type ferromagnets [equations (19) and (20)] and we have confirmed the validity of the approach by analyzing experimental data on nanocrystalline cobalt. The magnetic Guinier radius *R*_GSM_ depends on both the nuclear grain (anisotropy-field) microstructure and on the magnetic interactions (exchange-stiffness constant, saturation magnetization, applied field). It can be quite generally determined from the analysis of the magnetic field-dependent spin-misalignment SANS cross-section, which is obtained by subtracting the nuclear and magnetic scattering in the saturated state from data at lower fields. This method is easily applicable to magnetic materials using unpolarized neutrons.

## Supplementary Material

Additional neutron data and an error estimation. DOI: 10.1107/S2052252519016439/ti5016sup1.pdf
