# Supplementary material for: Magnetic Guinier law
Source: IUCrJ. 2020 Jan 1;7(Pt 1):136–42. doi: 10.1107/S2052252519016439 (PMC6949603; doi:10.1107/S2052252519016439)
Supplement: Supplementary file 1 [file m-07-00136-sup1.pdf]

IUCrJ

Volume 7 (2020)

Supporting information for article:

Magnetic Guinier Law

Andreas Michels, Artem Malyeyey, Ivan Titov, Dirk Honecker, Robert Cubitt, Elizabeth Blackburn and Kiyonori Suzuki

Supplemental Material to "Magnetic Guinier Law"

Andreas Michels,* Artem Malyeyev, Ivan Titov, and Dirk Honecker
Physics and Materials Science Research Unit,
University of Luxembourg, 162A Avenue de la Faïencerie,
L-1511 Luxembourg, Grand Duchy of Luxembourg

Robert Cubitt
Institut Laue-Langevin, 71 avenue des Martyrs, F-38042 Grenoble, France

Elizabeth Blackburn
Division of Synchrotron Radiation Research,
Department of Physics, Lund University, SE-22100 Lund, Sweden

Kiyonori Suzuki
Department of Materials Science and Engineering,
Monash University, Clayton, Victoria 3800, Australia

Abstract

In this Supplemental Material we provide additional neutron data and an error estimation in support of the above paper.

* Corresponding author: andreas.michels@uni.lu

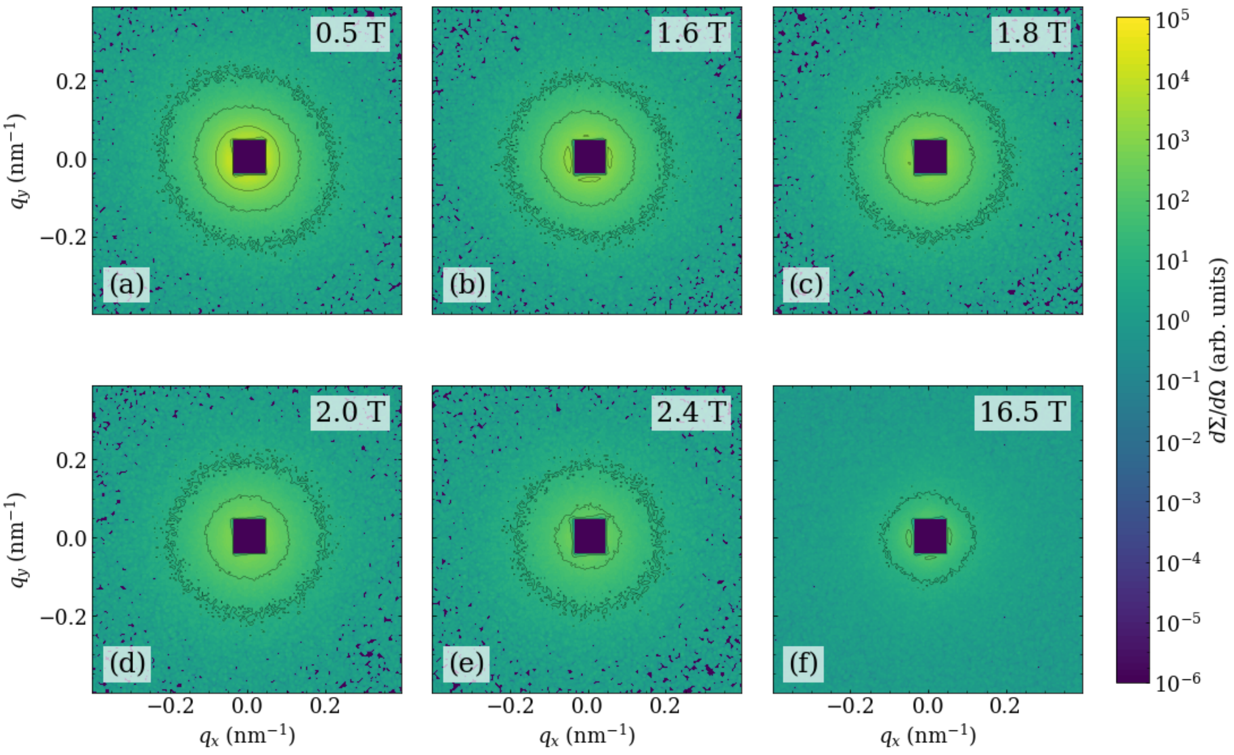

FIG. 1. Two-dimensional unpolarized total (nuclear and magnetic) SANS cross section $d\Sigma/d\Omega$ of nanocrystalline Cobalt at selected applied magnetic fields $\mathbf{H}_0$ (see insets) (logarithmic color scale). $\mathbf{H}_0 \parallel \mathbf{e}_z$ is applied parallel to the wave vector $\mathbf{k}_0$ of the incident neutrons. The average crystallite size of the Cobalt sample is $D = 9.5 \pm 3.0\,\mathrm{nm}$ [1].

---

[1] J. Weissmüller, A. Michels, J. G. Barker, A. Wiedenmann, U. Erb, and R. D. Shull, Phys. Rev. B **63**, 214414 (2001).

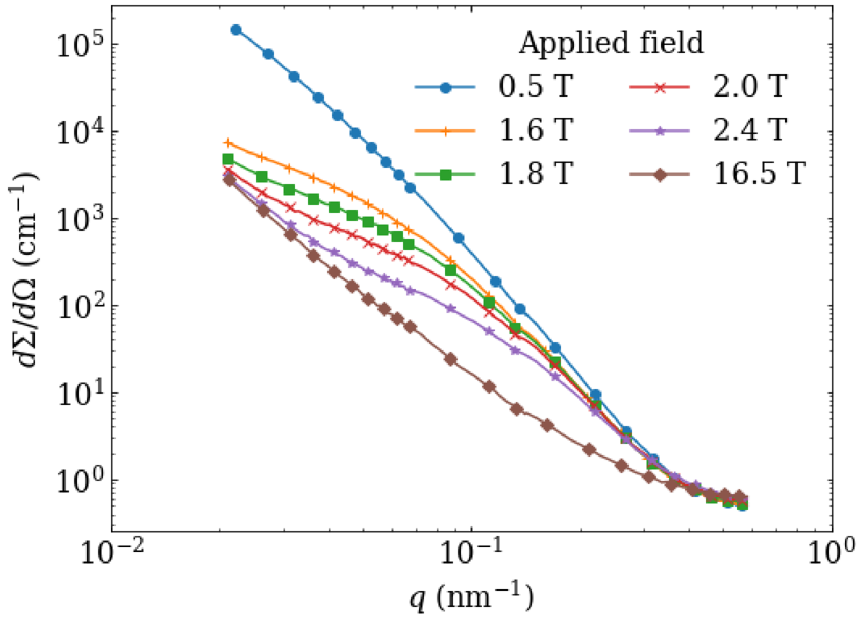

FIG. 2. 2π-azimuthally-averaged $d\Sigma/d\Omega$ data from Fig. 1 (log-log scale). For the clarity of presentation, the number of data points has been reduced.

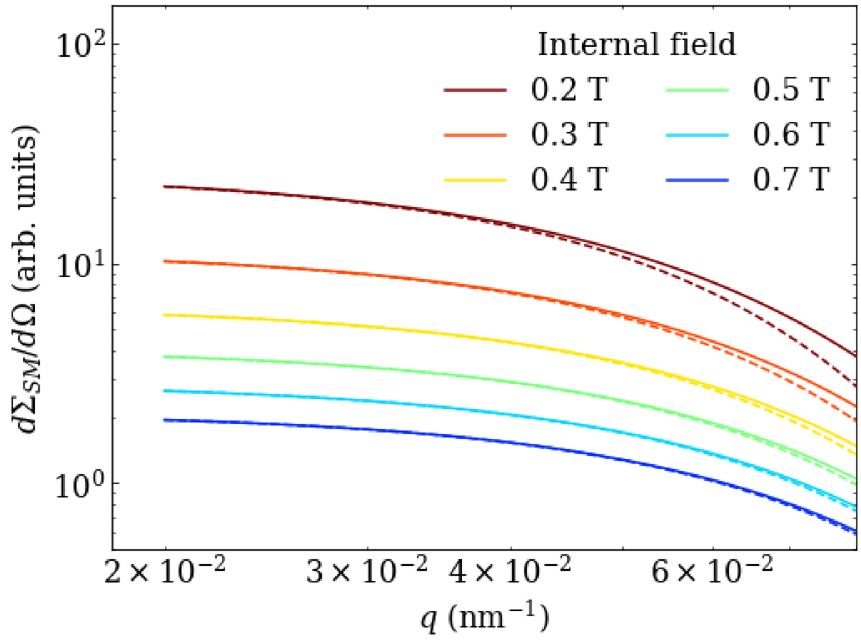

FIG. 3. Comparison between $d\Sigma_{SM}/d\Omega$ (solid lines, Eq. (7) in the paper) and the Guinier approximation [dashed lines, Eqs. (19) and (20)] at selected internal-field values (see inset) (log-log scale). The following materials parameters were chosen: $R_{GH} = 20.5\,\mathrm{nm}$, $A = 1.5 \times 10^{-11}\,\mathrm{J/m}$, $\mu_0 M_s = 1.80\,\mathrm{T}$.

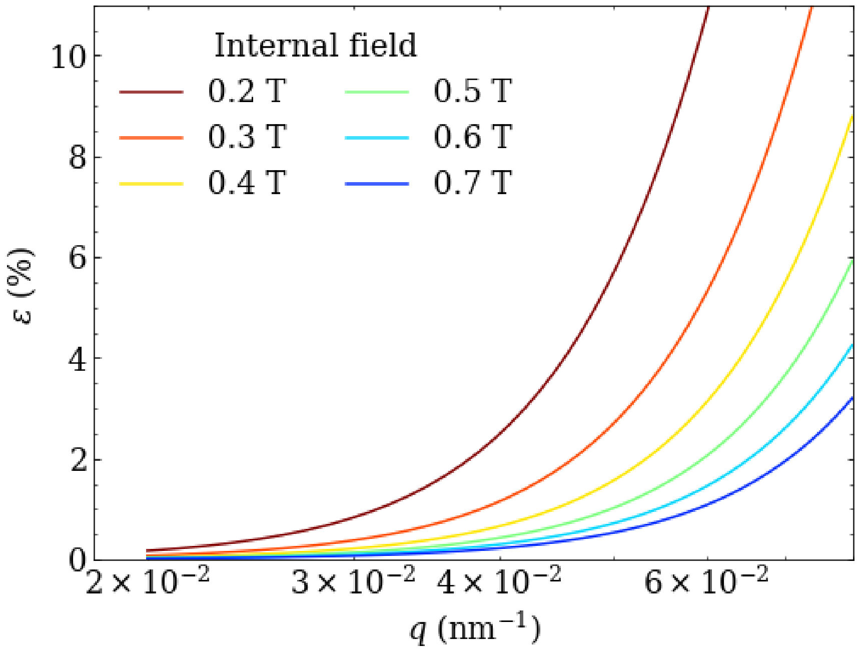

FIG. 4. Relative error of the Guinier approximation. Plotted is the quantity $\epsilon(q, H_i) = \sqrt{(f - f_G)^2}/f$, where $f = d\Sigma_{SM}/d\Omega$ (Eq. (7) in the paper) and f_G is the Guinier approximation [Eqs. (19) and (20)]. The following materials parameters were chosen: $R_{GH} = 20.5\,\mathrm{nm}$, $A = 1.5 \times 10^{-11}\,\mathrm{J/m}$, $\mu_0 M_s = 1.80\,\mathrm{T}$.
